# Electrical Diagnosis Techniques for Power Transformers: A Comprehensive Review of Methods, Instrumentation, and Research Challenges

**DOI:** 10.3390/s25071968

**Published:** 2025-03-21

**Authors:** Peter Mwinisin, Alessandro Mingotti, Lorenzo Peretto, Roberto Tinarelli, Mattewos Tefferi

**Affiliations:** 1 Department of Electrical, Electronic, and Information Engineering (DEI), University of Bologna, 40136 Bologna, Italy; peter.mwinisin@unibo.it (P.M.); lorenzoperetto@unibo.it (L.P.); roberto.tinarelli3@unibo.it (R.T.); 2 G&W Electric Co., Bolingbrook, IL 60440, USA; mtefferi@gwelec.com

**Keywords:** electrical diagnostics, condition monitoring, power transformer, instrumentation, partial discharge measurement, frequency response analysis (FRA), tan delta, dielectric response analysis, artificial intelligence

## Abstract

This paper serves as a comprehensive “starter pack” for electrical diagnostic methods for power transformers. It offers a thorough review of electrical diagnostic techniques, detailing the required instrumentation and highlighting key research directions. The methods discussed include frequency response analysis, partial discharge testing, dielectric dissipation factor (tan delta), direct current (DC) insulation resistance, polarization index, transformer turns ratio test, recovery voltage measurement, polarization–depolarization currents, frequency domain spectroscopy, breakdown voltage testing, and power factor and capacitance testing. Additionally, the paper brings attention to less-explored electrical diagnostic techniques from the past decade. For each method, the underlying principles, applications, necessary instrumentation, advantages, and limitations are carefully examined, alongside emerging trends in the field. A notable shift observed over the past decade is the growing emphasis on hybrid diagnostic approaches and artificial intelligence (AI)-driven data analytics for fault detection. This study serves as a structured reference for researchers—particularly those in the early stages of their careers—as well as industry professionals seeking to explore electrical diagnostic techniques for power transformer condition assessment. It also outlines promising research avenues, contributing to the ongoing evolution of transformer diagnostics.

## 1. Introduction

The electrical power system is a complex grid consisting of various interconnected components that work together to achieve the primary goal of generating and delivering electricity to end-users. This system includes assets such as conventional and distributed generators, transformers, transmission and distribution lines, switchgear, instrument transformers (ITs), protective devices, and other miscellaneous equipment [1,2]. Condition monitoring and diagnostic processes of these assets are critical for grid operators. To ensure the safe and reliable operation of the power system, continuous monitoring and assessment of power equipment—both online and offline—are essential [3,4]. Equipment failures within electrical networks can result in significant financial losses for power utilities. Therefore, the use of reliable assessment methods is crucial to accurately determine the condition and performance of system assets. Accurate diagnostics enable strategic asset management, optimizing acquisition techniques investment, maintenance costs, and operational efficiency [5]. Condition diagnosis methods encompass both traditional techniques and emerging approaches, particularly those leveraging artificial intelligence (AI) and machine learning (ML) models, for identifying failure mechanisms across various voltage levels [6,7].

The general procedure for condition monitoring and diagnosis of electrical assets is outlined in [8,9]. Regardless of the diagnostic technique employed, two primary processes are involved: data acquisition and data analysis. The first step involves gathering data, which can be achieved through equipment inspection or by conducting tests to measure specific parameters. Depending on the data’s nature or required sample size, tests may be continuous in real-time or one-time measurements. These tests or inspections can be performed either online or offline [10]. Some tests are only possible when the asset is in service or offline. However, due to the different dynamic behavior of electrical equipment in service and out of service, utility operators prioritize understanding the asset’s condition in both scenarios. To minimize equipment downtime during offline testing, efforts are underway to develop innovative strategies that enhance traditional offline data acquisition methods, allowing them to effectively capture data in real time during operation [11]. The nature of tests can vary, including electrical, chemical, physical, thermal, and mechanical forms. Chemical and electrical tests dominate the literature, while the other test categories are still being explored.

**Figure 1:**
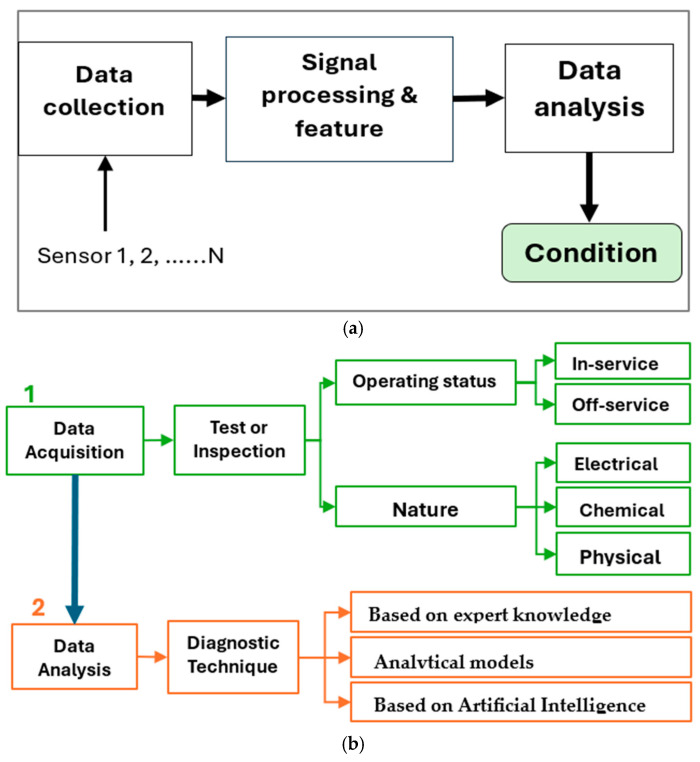
Overview of the general procedure for condition monitoring and fault diagnosis (**a**) [9] (**b**) [8].

In the second step, which involves analyzing the acquired data, various diagnostic techniques can be employed. These diagnostic or signal processing techniques are used to extract meaningful information from raw data and can be broadly categorized into three groups: AI-based analysis, analytical models, and expert judgment. While traditional diagnostic methods based on expert knowledge have yielded satisfactory results, there is increasing skepticism about their reliability. Expert judgment relies on experience and often involves interpreting charts, graphs, images, and other data forms to draw conclusions. In [8], the authors classified methods such as fuzzy logic and expert systems as forms of expert judgment in diagnosis. On the other hand, analytical models are grounded in the mathematical formulations of the system or equipment. While these models can yield more reliable results, any errors in the formulation can propagate throughout the analysis, potentially compromising accuracy. The rise of AI technologies has introduced innovative approaches for diagnosing faults in electrical equipment, such as neural networks, gray system theory, genetic algorithms (GAs), space vector models (SVMs), decision trees, deep belief networks (DBNs), and others. These AI-based techniques have significantly improved diagnostic accuracy and reliability, enhancing the ability to effectively identify and address faults [7,12,13]. Currently, the trend is shifting towards combining multiple AI-based methods or integrating expert knowledge with AI techniques to achieve even more reliable results. Figure 1 illustrates the general approach to electrical asset diagnosis as presented in the literature.

### Importance of Power Transformer Diagnosis

The condition monitoring and timely fault diagnosis of power transformers is critical to the reliability and security of power supply to end users. Some benefits include the follow:

- Elimination of high economic losses associated with partial or total repair [14];
- Increase in the uptime of equipment;
- Improvement in the quality of equipment performance and avoid untimely degradation [15];
- Reduced outages in the production and distribution of electricity;
- Prevention of conditions that put operators in risk [9];
- Increase in the life expectancy of the assets.

Several other benefits can be derived from the effective fault diagnosis. In its absence, operators may encounter negative consequences due to the lack of accurate and continuous monitoring of their equipment.

While there are numerous review papers on power transformers in the literature, they often lack specificity. For instance, electrical, acoustics and ultra-high-frequency acquisition methods for partial discharges were briefly reviewed in [16]. Similarly, both traditional and emerging condition monitoring were put together in a brief review in [17]. In an attempt to diagnose and detect faults on a power transformer following an earthquake, the authors of [18] reviewed and applied various diagnostic techniques. The advances of computer applications in both traditional and recent diagnostic techniques have been extensively studies in [19]. Also, the various methods for diagnosing power transformers, including electrical, chemical, acoustic, and other techniques, have been combined and reviewed in [20,21].

This paper, however, exclusively focuses on electrical diagnostic techniques for power transformers. It discusses detection methods, the parameters/variables detected, and the instrumentation required for each test conducted on the asset.

**Figure 2:**
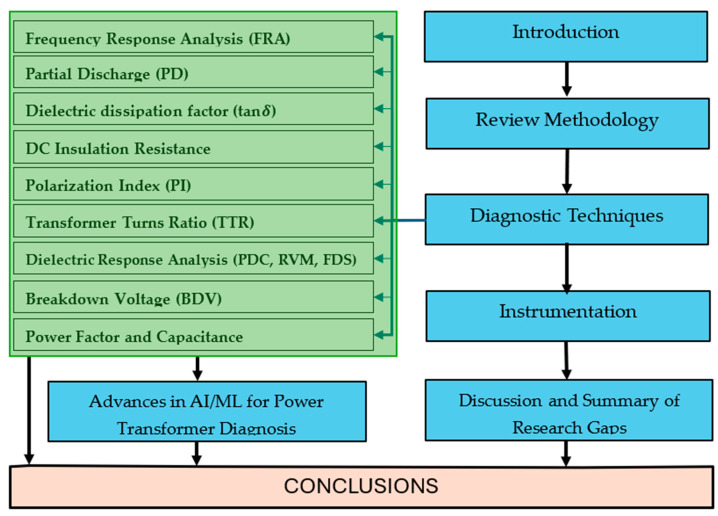
Flowchart illustrating the organization of the paper.

The structure of the paper is as follows: Section 1 presents the introduction, providing an overview of fault diagnosis, the taxonomy of the process, and the importance of timely fault detection. Section 2 outlines the methodology used for the literature review, including the criteria for paper selection and the potential biases of the process. Section 3 delves into the main electrical diagnostic techniques for power transformers. Each method is discussed in detail, followed by a summary of the techniques presented in a table. In Section 4, the advances in artificial intelligence/machine learning for power transformers is discussed. Section 5 examines the instrumentation needed for the various diagnostic strategies. Section 6 presents a discussion of the review’s results, highlighting key trends and research directions in diagnostic techniques. Finally, Section 7 concludes the paper, summarizing the main findings and offering recommendations for future research. In Figure 2, the flowchart of how the paper is organized is presented.

## 2. Review Methodology

The Preferred Reporting Items for Systematic Reviews and Meta-Analyses (PRISMA) guidelines [22], one of the most widely adopted standards for reporting systematic reviews and meta-analyses, have been followed for this review. The 27-item checklist provided by PRISMA 2020 serves as a guide for presenting this review. The data sources for this review include IEEE Xplore, Scopus, and Google Scholar databases. Additionally, papers from the 10th International Conference on Condition Monitoring and Diagnosis (CMD 24) held in Korea were incorporated into the review. For the IEEE Xplore, Google Scholar, and Scopus databases, an advanced search was conducted using relevant keywords to identify papers pertinent to this review. The primary keywords used in this search were “Diagnos*” and “condition* monitoring”, where the asterisk (*) accounts for variations in the keywords. This ensures that synonyms such as diagnosis, diagnosing, diagnostic, condition monitoring, conditional monitoring, and related terms are included in the search results, thereby capturing the terminological variations used by different researchers. To perform the advanced search, the two primary keywords were combined with logical operators and the specific electrical asset of interest. The logical operators used in this search were AND, OR, and NOT. The AND operator combined the asset name with the keywords, while the OR operator was used to include synonyms of the keywords. The NOT operator helped exclude undesired terms. For example, when focusing on data related to power transformer, the NOT operator was employed to filter out distribution transformer and instrument transformers from the search results. The general command for the advanced search is as follows:

(“All Metadata”: Power transformer) AND (“All Metadata”:diagnos*) OR (“All Metadata”: condition* monitoring) NOT (“All Metadata “: words or phrase to exclude)

### Data Segregation and Account for Bias

To refine the search results, several filters were applied. First, to focus on the trends in electrical diagnostic methods over the last decade, the year filter was set to include only papers published between 2014 and 2024. After obtaining the initial results, further screening was conducted. Additionally, the advanced search was performed solely on document titles, rather than across all metadata. This ensured that only papers with titles containing the desired keywords were retrieved, allowing for a more focused selection and excluding papers that merely mentioned the keywords in passing. Furthermore, for each individual search within this time frame, papers were further excluded based on their abstracts. Any papers that discussed non-electrical diagnostic methods (e.g., chemical methods) were removed from the results. The updated advanced search algorithm is as follows:

(“Document Title”: Power transformer) AND (“Document Title”: diagnos*) OR (“Document Title”: condition* monitoring) NOT (“Document Title”: words or phrase to exclude): 2014–2024

**Figure 3:**
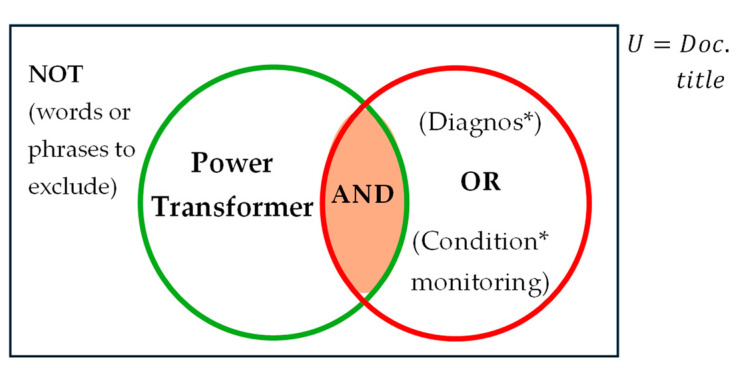
Graphical representation of the advanced search algorithm.

Although the data sources for this review are heterogeneous, some biases may still exist. Given that Scopus is one of the largest repositories of scientific and technical research papers, it was chosen as the primary search database. One advanced search was conducted in this database using the search command outlined above. To minimize the potential bias of overlooking relevant papers published in other reputable journals, supplementary searches were performed in the other databases mentioned. These supplementary searches were designed to identify specific papers. For example, to explore a particular diagnostic technique, a targeted search was conducted. Additionally, supplementary searches were used to locate review papers on condition monitoring for power transformer. In these cases, the advanced search algorithm was slightly adjusted to include the desired terminologies using the AND logic operator. The general advanced search algorithm is depicted in Figure 3, where the shaded intersection represents all retrieved papers.

## 3. Power Transformer Diagnosis Techniques

Power transformers are core assets of the electrical grid; hence their continual operation cannot be compromised. The unexpected failure of one power transformer implies power outage to sometimes hundreds or thousands of users. Several diagnosis techniques have been presented in the literature for power transformers ranging from chemical and electrical methods to acoustic techniques [23,24]. Some major electrical diagnosis techniques for power transformers are discussed below.

### 3.1. Frequency Response Analysis

**Figure 4:**
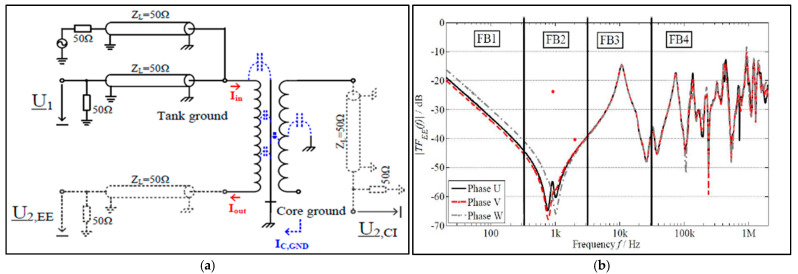
(**a**) FRA measurement circuit [30]; (**b**) typical FRA profile with frequency band [30].

Frequency response analysis (FRA) is widely considered the most accurate and advanced method for diagnosing changes or deformations in the mechanical components of transformers, including the core, windings, and clamping structures [25,26]. Depending on the input signal, FRA can be classified into Sweep FRA (SFRA) or Impulse FRA (IFRA). In SFRA, a sweep sinusoidal signal is applied to the transformer, typically with a frequency range between 10 Hz and 1 MHz [27]. In contrast, IFRA involves applying an impulse voltage signal, with the frequency response derived by performing a Fast Fourier Transform (FFT) on the recorded output signals [28,29]. SFRA is commonly performed offline while IFRA is an offline and online technique [28]. The two primary test methods used for measuring the transfer function (FRA) of power transformers are the end-to-end transfer function measurement, denoted as ${TF}_{EE}(f)$, and the capacitive inter-winding transfer function measurement, represented as ${TF}_{C1}(f)$ [30]. The measurement setup and typical profiles of measurement are shown in Figure 4. The reference international standard dedicated to FRA tests are IEC 60076-18 [31] and IEEE C57.149-2012 [32].

The end-to-end transfer function measurement, ${TF}_{EE}(f)$, and the capacitive inter-winding transfer function measurement, ${TF}_{C1}(f)$ can be computed as follows:

(1) $${TF}_{EE}(f)=\frac{U_{2,EE}}{U_{1}}$$

(2) $${TF}_{C1}(f)=\frac{U_{2,C1}}{U_{1}}$$

Despite its widespread use, several studies have reported challenges related to the uncertainty in interpreting FRA signatures and correlating them with specific fault types [24,29,33]. To improve the interpretation of FRA signatures, various approaches have been proposed, including statistical methods [34] and AI and machine learning techniques. For example, in [35], the authors employed F-test and T-test statistical tools to distinguish between normal and abnormal responses in FRA signatures. Additionally, the authors of [29,36] proposed a method that combines both the magnitude and phase of the FRA signature in a polar plot to enhance interpretation accuracy. Furthermore, AI, particularly neural networks, has been suggested by [37] to improve the interpretation of FRA signatures. However, research gaps remain in the areas of FRA signature interpretation, accurate fault identification using FRA signatures, and limited online FRA applications. The challenge of injecting test signals into a transformer while it is online has constrained research on online FRA, as noted by [28]. The authors suggest further research in this direction.

### 3.2. Partial Discharge

**Figure 5:**
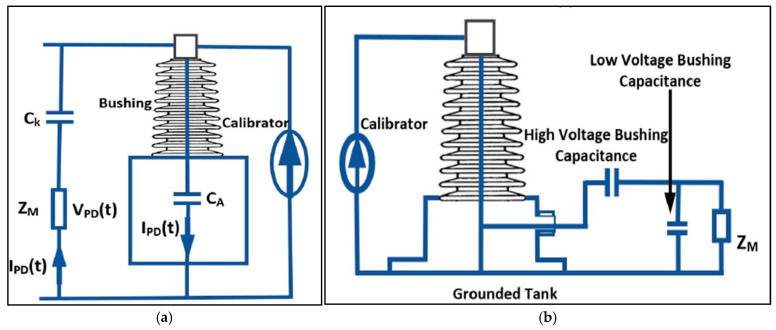
Partial discharge measurement setup [43]. (**a**) Employing external coupling capacitor (**b**); coupling from busing taps.

Partial discharge (PD) measurements can be performed through electrical, acoustic, and ultra-high-frequency (UHF) acquisition techniques depending on the physical PD phenomenon of interest [38,39]. In the context of electrical methods, which is the focus of this paper, PD measurements can be performed in three primary ways: through an indirect circuit using an external coupling capacitor, employing inductive sensors such as High-Frequency Current Transformers (HFCT), and coupling PD signals from bushing taps [40,41]. The electrical method of PD measurement based on the IEC 60270 standard [42] employing an external coupling capacitor is depicted in Figure 5a [43]. This method is limited to laboratory settings because of the bulky nature of the high-voltage coupling capacitor. However, if a bushing tab is available, online measurement can be employed using the setup Figure 5b [43,44].

Although the electrical PD measurement outlined in the IEC 60270 standard [42] is considered the most sensitive among the three methods mentioned here [45], it has several limitations, such as being suitable only for laboratory or factory settings and being susceptible to electromagnetic disturbances especially the online method [46,47]. As a result, further research is needed to address these challenges. Over the last decade, most of the electrical PD research has focused on PD detection, measurement, and classification. Electrical methods for PD localization typically model the PD phenomenon within insulation as an equivalent circuit, often using a winding system model. A classic example of this is the abc-circuit model for a gas-filled void in insulation [48]. Electrical PD localization techniques are detailed in [16]. These models are not widely adopted in practical applications, leading to greater reliance on other well-established localization methods such as acoustic and UHF detection, and more recently, the use of machine learning algorithms [49,50,51]. Researchers strongly recommend hybrid methods for the detection, measurement, classification, and localization of PD sources to increase accuracy [52]. In [53], the authors proposed a combined in-oil PD sensor which is based on AE and UHF methods for PD detection. According to [46], the future of research on PD activity is towards advancing the understanding of PD signal propagation within transformers, optimizing sensor design, and developing accurate charge calibration techniques.

### 3.3. Dielectric Dissipation Factor/Tan Delta

**Figure 6:**
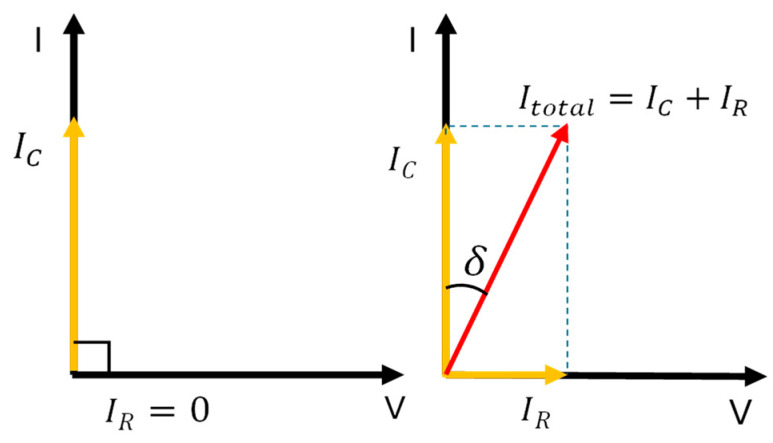
Relationship between I_R_ and I_C_. (**Left**): Perfect in insulation; (**Right**): actual insulation.

Tan delta, also known as the dielectric dissipation factor, is a critical parameter used to assess the condition and reliability of insulation systems in electrical equipment, such as power transformers. Ideally, insulating materials, or dielectrics, are expected to behave as perfect, lossless capacitors [54,55]. In this ideal scenario, the voltage and current are in a 90-degree phase shift, with the current through the insulation being purely capacitive. In practical insulation systems, however, a resistive loss current ($I_{R}$) flows in phase with the voltage [56]. The magnitude of this resistive component is influenced by factors such as moisture, aging, and the presence of conductive impurities in the insulating oil, while the capacitive current ($I_{C}$) is frequency-dependent [57]. The presence of contaminants reduces the insulation’s resistance, resulting in an increase in resistive current and a deviation from the ideal capacitive behavior. As a result, the phase shift between voltage and current becomes less than 90 degrees due to the combined effects of $I_{R}$ and $I_{C}$ on the total current angle. The extent of this deviation, known as the loss angle (δ), reflects the level of contamination and serves as an indicator of the insulation’s quality and reliability. This loss angle is carefully measured and analyzed, with the tangent of the loss angle (tan (δ)) representing the ratio of resistive current to capacitive current [58]. By conducting a tan delta test, it becomes possible to evaluate the insulation’s resistance and overall health effectively. The classical setup for tan (δ) measurement is outlined in [59]. The greater is the value of tan (δ), compared to the value stipulated in the standards, the lower is the quality of the transformer insulation system being tested [58]. In Figure 6, the relationship between capacitive and resistive current for perfect and actual insulation is presented [58]. The IEC 60851-5:2008 standard [60] is the international document relating to the tan (δ) test. It provides specific guidelines for the measurement of tan (δ).

(3) $$\tan\left(\delta \right)=\frac{\left|I_{R}\right|}{\left|I_{C}\right|}$$

While tan (δ) is a method for testing the insulation condition of various electrical assets, this method has predominantly been used for diagnosing the insulation of cables [61,62]. From the bibliography extracted using the advanced search algorithm explained above, it has been observed that few papers have been published in the last decade on the use of the tan (δ) technique for assessing the insulation of power transformers. Notable uses of tan (δ) for transformer insulation diagnosis include [56]. In this research, tan (δ) was used alongside other diagnostic methods to assess the insulation integrity of a power transformer over 30 years old at the Belawan Combined Cycle Power Plant. Similarly, the authors in [63] conducted tan (δ) tests on the windings and bushings as part of a comprehensive electrical diagnostic procedure to identify the cause of a breakdown in the T-phase bushing of a 500/150 kV inter-bus transformer (IBT) at the Cirata Extra-High-Voltage (EHV) Substation. While the tan (δ) test is a simple and effective method for evaluating insulation condition, it is typically used in conjunction with other diagnostic techniques to support informed decision making. Additional example can be found in [50]. According to [64], the main challenges regarding the tan (δ) assessment of electrical insulation can be grouped into four broad categories:

- Transportation: A key challenge highlighted is the large size and weight of measurement devices, which limit transportation flexibility. End users prefer lightweight and compact designs to reduce transportation, handling, and storage costs.
- Testing or measurement: Another challenge in dielectric loss measurement is selecting the right measuring device, as post-processing availability and reliable, repeatable results are crucial for electrical engineers and researchers to take accurate actions.
- Processing the results: After measurement, analyzing and post-processing the results is essential, with accuracy being a primary concern.
- Standards and others: Testing procedures and experimental results should adhere to international standards to assess the test object’s condition. Strict adherence to these standards poses a challenge to manufacturers and researchers.

### 3.4. DC Insulation Resistance

The insulation resistance (IR) test is a vital diagnostic tool used to evaluate the condition of electrical insulation, specifically between windings and ground or between two windings. This test measures the resistance to leakage current that flows through the insulation or along its surface. A decrease in IR often indicates contamination, moisture ingress, or deterioration of the insulation material [18,65]. The IR test typically involves applying a high DC voltage to induce a small current that flows through and along the insulation. The total current consists of four components: dielectric absorption current (polarization current), conduction current, leakage current, and capacitive charging current. Among these, leakage current is the most critical parameter for assessing insulation integrity, as it directly reflects the health of the insulation system [63,66].

Several factors influence IR measurements, including temperature, humidity, moisture content, air pollutants, and surface contamination such as dirt on bushings or insulators [67]. IR is measured using a megger (megaohmmeter), and is commonly expressed in megaohms [68]. The test is usually performed with a DC voltage ranging from 500 to 10,000 V, depending on the test requirements (refer to Table 1) [56,68]. The IR value is calculated by dividing the applied voltage by the leakage current, with corrections made for temperature variations, typically up to 40 °C [56,69].

The testing procedure follows established standards such as IEC 60076-3:2013 [70]. It is essential for identifying faults like open windings, shorted turns, and more subtle degradation within the transformer’s insulation system. By evaluating IR, asset managers can determine the current health of the insulation and predict potential future failures, enabling proactive maintenance strategies [71].

IR testing is commonly used in combination with other diagnostic methods, particularly the Polarization Index (PI), which is an extension of the IR test [71,72]. In [73], the authors employed a statistical approach to assess and classify the insulation condition of power transformers. They introduced a novel equation to calculate and assign condition scores based on historical IR test data collected from 237 power and distribution transformers. The study concluded that integrating maintenance engineering criteria with computational optimization techniques resulted in an effective diagnostic method for transformers. The impact of through-fault current—defined as current resulting from a power system fault occurring outside the protected section but flowing through the protected area—on the IR of power transformers in different substations was also investigated in [68]. The study found that through-fault current generally does not affect the IR of transformers due to its random and transient nature. However, it was also observed that through-fault current can negatively impact the primary insulation of the transformer’s primary winding.

Areas for further research on insulation resistance include evaluating the influence of environmental variables such as humidity and temperature on the accuracy of test results, as well as investigating the effects of aging and wear on the insulation resistance of different insulating materials.

### 3.5. Polarization Index

The PI measurement is used for assessing the condition of insulation in electrical equipment, including transformers, cables, and electric machines [74]. PI is defined as the ratio of IR measured at 10 min (${IR}_{10min}$) to that measured at 1 min (${IR}_{1min}$) under a constant test voltage. This ratio provides insights into the cleanliness, dryness, and overall health of the insulation system [73].

(4) $$PI=\frac{{IR}_{10min}}{{IR}_{1min}}$$

Similarly to IR testing, the PI test evaluates an insulation system’s ability to resist leakage currents, providing a reliable indicator of contamination and moisture levels. Clean, dry insulation exhibits increasing resistance over time, resulting in higher PI values. In contrast, contaminated or moisture-laden insulation shows minimal resistance growth, leading to lower PI values. PI values are typically categorized as follows: 1.5–2.0 indicates dry insulation, 1.0–1.5 suggests dirty or wet insulation, and values below 1.0 indicates severe contamination requiring immediate repairs [57]. The minimum PI value depends on the thermal class rating of the insulating material used as established in Table 2.

The primary purpose of PI testing is to assess whether electrical equipment is fit for operation or requires further evaluation, such as overvoltage testing. During the test, continuous IR readings are recorded over a 10 min period under a constant test voltage, allowing the resistance to stabilize. A rapid increase in resistance indicates clean, dry insulation, while slow increases or stagnant values suggest contamination, moisture, or insulation degradation [56]. For power transformers, PI testing can be conducted between a phase and ground or between different phases. The various testing modes are summarized in Table 3.

Although PI testing is a quick and convenient method for evaluating insulation condition, it has notable limitations. It is ineffective in assessing insulation aging or degradation caused by long-term mechanical stress [68]. As a result, PI testing is often complemented by advanced diagnostic methods such as FRA and dissolved gas analysis (DGA) to provide a more comprehensive assessment of insulation integrity. While PI naturally complements the IR test, it has also been used alongside other electrical diagnostic techniques, as demonstrated in [18], to support comprehensive failure analysis.

Future research could focus on the impact of ambient conditions—such as temperature, humidity, and pressure—and test parameters like voltage levels and polarity on PI results. Additionally, exploring hybrid diagnostic approaches that combine PI with other advanced techniques may enhance diagnostic accuracy and reliability.

### 3.6. Transformer Turn Ratio

Transformer turn ratio (TTR) testing is a well-established diagnostic method used to evaluate transformer performance, detect winding defects, and assess insulation integrity. The TTR represents the ratio of the number of turns in the primary winding to those in the secondary winding, providing critical insights into winding balance and potential defects that may affect output voltage. The test verifies the accuracy of the transformer turn ratio and helps identify issues such as tap-changer malfunctions, shorted turns, faulty connections, and open windings. Regular TTR testing enables condition monitoring over time, supports quality verification, and facilitates early defect detection [75].

TTR testing involves applying a low AC voltage to measure the ratio between the primary and secondary windings, with tests conducted at each tap position of the transformer [76]. The process typically starts with a low-voltage application—often around 100 V—to minimize the risk of insulation breakdown, gradually increasing to the rated voltage if no significant deviations are detected [77]. Deviations exceeding 0.5% may indicate issues such as insulation failure, short circuits, and open turns. Testing across all tap positions is essential for identifying irregularities, ensuring the comprehensive assessment of winding conditions [78].

The TTR test setup typically includes at least two high-voltage leads and two low-voltage leads. Single-phase and three-phase TTR models are available to suit different applications. Single-phase models are ideal for transformers with low turn ratios, requiring separate tests for each phase, while three-phase models are designed for high-ratio transformers, including power, instrument, and distribution transformers [79]. TTR tests typically follow a two-step process involving the measurement of voltage ratio of the transformer ($V_{TTR})$ between primary ($V_{p}$) and secondary ($V_{s}$) windings, followed by the calculation of the percentage error (${∆}_{ratio}\%$) between measured ratio ($V_{r\_m}$) and nameplate values ($V_{r\_np}$). IEEE standards specify that this error should not exceed 0.5% [34]. TTR and the percentage error are given in Equations (2) and (3). The standards adopted for TTR test are IEEE C57.12.90-2021 [80] and IEC 60076-1:2013 [81].

(5) $$V_{TTR}=\frac{V_{p}}{V_{s}}=\frac{N_{p}}{N_{s}}$$

(6) $${∆}_{ratio}\%=\left|\frac{V_{r\_m}-V_{r\_np}}{V_{r\_np}}\right|$$

While TTR testing offers significant benefits, including verifying transformer design specifications, detecting winding defects, and monitoring tap-changer performance, it is rarely used as a standalone diagnostic tool. Instead, it is often combined with other techniques, particularly SFRA, to enhance diagnostic accuracy. In [75], the authors integrated TTR with SFRA to analyze a transformer suspected of imminent failure. The results showed a linear correlation between TTR and SFRA, indicating that both methods effectively complement each other in diagnosing transformer faults. Similarly, in [34], the combined use of TTR and SFRA demonstrated that SFRA enhances TTR’s diagnostic capabilities by detecting inter-turn faults through frequency response variations and statistical indicators.

Given the complementary nature of TTR and SFRA, a potential research gap exists in developing a standardized hybrid diagnostic framework that integrates both methods for improved accuracy. Although TTR has been extensively combined with other diagnostic techniques, such as in [18] for the post-seismic fault diagnosis of power transformers, further research is recommended to refine and expand these integrations. Additionally, the application of machine learning and AI tools presents a promising avenue to automate and enhance TTR data analysis, particularly for efficiently processing large datasets and identifying subtle fault patterns.

### 3.7. Dielectric Response Analysis

Dielectric response analysis (DRA) is an advanced technique used to evaluate the moisture content in the oil–paper insulation of transformers. Moisture generation is influenced by various factors, including organic acids, gases, humidity, ambient temperature, and oxygen levels. Traditional methods, such as Karl Fischer Titration (KFT) and the Piper–Fessler isothermal model, can measure moisture directly but come with practical limitations [82]. KFT requires paper samples from multiple locations within the transformer, making it unsuitable for on-site testing. Similarly, the accuracy of the Piper–Fessler method depends heavily on the placement and adequacy of moisture sensors inside the transformer tank. Moreover, temperature fluctuations can disrupt moisture equilibrium between the oil and paper, reducing the reliability of equilibrium charts and introducing significant measurement errors [83]. To overcome these challenges, recent studies have increasingly focused on spectrum-based methods for evaluating moisture content and aging by-products in transformer insulation systems [84]. In this context, DRA has emerged as a reliable and non-destructive alternative. DRA techniques, such as recovery voltage measurement (RVM), polarization and depolarization current (PDC) analysis, and frequency domain dielectric response (FDDR) analysis, offer direct, accurate, and non-invasive assessments of insulation condition [84,85].

#### 3.7.1. Recovery and Return Voltage Measurement

Recovery voltage measurement (RVM) and return voltage measurement (RVM) are both dielectric response analysis techniques used to diagnose aging and moisture content in power transformers. Although these methods differ in their underlying principles, they are often used interchangeably in literature.

Starting with recovery voltage measurement, this technique involves the application of a DC voltage and is designed to measure the interfacial polarization spectrum using a recovery voltage meter [84,86]. This spectrum is highly sensitive to moisture and aging, making RVM an effective diagnostic tool for assessing transformer insulation integrity. The RVM process begins with the application of a DC voltage ($U_{P}$) typically in the kilovolt range for a duration of time called charging/polarization time ($t_{c}$) (commonly 900 or 1800 s). After this charging period, the test object is short-circuited for a discharge period $t_{d}$. This process is repeated, increasing both the charging period ($t_{c}$) and the short-circuiting period ($t_{d}$) at each cycle and maintaining a fixed ratio of $t_{c}/t_{d}=2$ throughout the measurement sequence.

**Figure 7:**
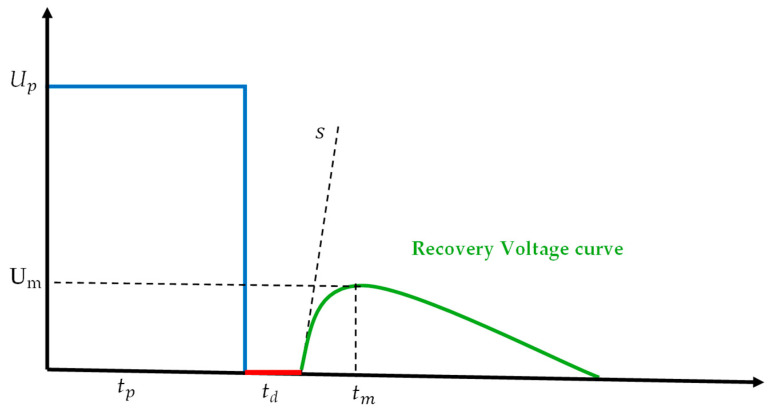
Recovery voltage spectrum [88].

Once the short circuit is removed after each cycle, a recovery voltage develops across the terminals of the test object, rising to a peak before gradually declining. This voltage results from remanent charges retained within the insulation medium and is directly related to the insulation’s quality [87]. A series of recovery voltage measurements, conducted with increasing charging times, generates the polarization spectrum, which provides valuable insights into the moisture content and aging processes within transformer insulation. The shape and characteristics of the voltage curve contain critical diagnostic information for evaluating insulation health as depicted in Figure 7 [84,88].

**Figure 8:**
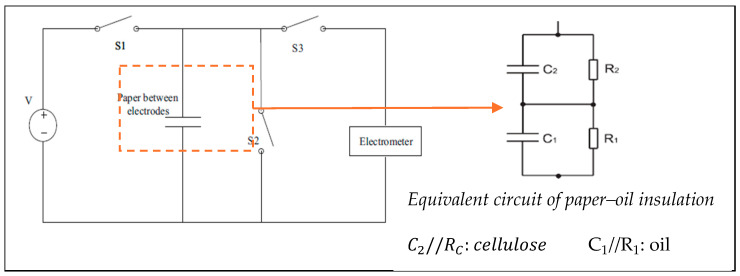
Circuit for recovery voltage measurement [57,88].

As depicted in Figure 8, to apply the DC voltage on the test object, switch 1 ($S_{1}$) is first closed for $t_{c}$, during which the capacitor is charged. After closing $S_{1}$ and opening $S_{2}$, a short circuit is created and the capacitor discharges for a period $t_{d}$. After the short circuit is removed, the return voltage waveform generated is as depicted in Figure 5.

The key features of the polarization spectrum are the maximum amplitude of the voltage and its corresponding time constant. The RC branches representing the cellulose and oil yield two distinct time constants, $\tau_{1}$ and $\tau_{2}$, where their ratio is given as $\lambda =\tau_{2}/\tau_{1}$. The maximum voltage $U_{m}$, the time to reach it $t_{m}$, and the initial slope of the curve s can be computed using the time constants and $U_{s}$, which depends on the geometric dimensions of the insulation system. The relationships are as follows:

(7) $$s=\frac{U_{s}}{\tau_{1}}\left(\frac{\lambda -1}{\lambda }\right)$$

(8) $$U_{m}=U_{s}\left(\lambda^{1/\left(1-\lambda \right)}-\lambda^{\lambda /\left(1-\lambda \right)}\right)$$

(9) $$t_{m}=\tau_{1}\left(\frac{\lambda }{\lambda -\lambda }\right)\ln\lambda$$

The insulation condition of the transformer can be diagnosed by comparing the generated recovery voltage curve with the ideal curve derived from the Maxwell model [88,89]. Additionally, the time constants ($\tau_{1}$ and $\tau_{2}$), along with the reliability factor r (defined as the logarithmic product of the two time constants in (7)) can serve as valuable diagnostic tools. It is important to note that in the case of uniform insulation, only a single time constant would be observed.

(10) $$r=\log\tau_{1}\times \log\tau_{2}$$

The following can be considered a rule of thumb:

r>5: New/good insulation;

r<2.5: Severe aging and degradation, high water content.

The key distinction between recovery voltage measurement (RVM) and return voltage measurement lies in the application of the DC voltage and the number of cycles involved. In return voltage measurement, unlike the previously discussed recovery voltage method, a single high voltage, typically in the range of several kilovolts, is applied. Once the short circuit on the tested object is removed, the return voltage generated between the measuring electrodes can rise to several hundred volts. This characteristic makes return voltage measurement particularly resistant to interference from external electric fields in the surrounding environment [88].

While recovery/return voltage measurement is effective in diagnosing power transformer insulation, it is not without its challenges. Comparative studies between RVM and other techniques, such as FRA, have been rare. A potential research direction could involve conducting systematic comparative studies to evaluate the strengths, limitations, and specific applications of RVM in relation to other diagnostic methods. Another promising avenue could explore how RVM complements other techniques for a more comprehensive diagnostic approach. RVM is particularly useful for assessing the moisture content and aging of oil–paper insulation. Factors like transformer loading levels, temperature, and other environmental conditions contribute to moisture generation. An interesting area for further research could be evaluating the effectiveness of the RVM technique under varying load and temperature conditions. Additionally, it has been noted that distinguishing the separate effects of moisture and aging in RVM diagnoses is challenging. Developing a simpler method to address this issue would fill an important research gap.

#### 3.7.2. Polarization Depolarization Currents (PDCs)

**Figure 9:**
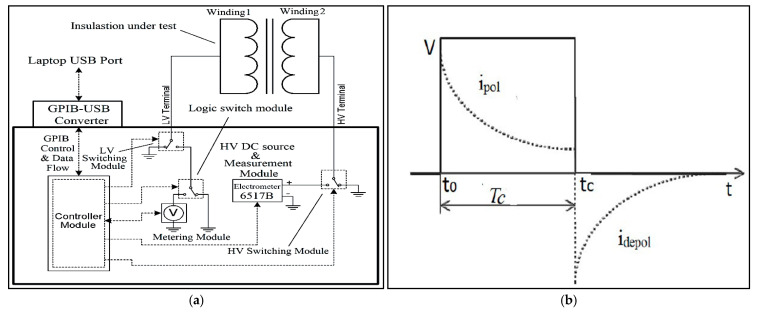
(**a**) PDC measurement test setup [88]; (**b**) typical trend of monotonically decreasing PDC profile [92].

Polarization depolarization current (PDC) testing is conducted in two phases: the polarization current measurement phase followed by the depolarization current measurement phase. During the polarization current measurement, a DC voltage, typically 1000 V, is applied to the insulating material being evaluated [90]. When exposed to this DC step field, the internal dipoles within the insulating material align with the direction of the applied field, initiating the polarization process and generating a polarization current through the material. In the depolarization phase, the supply terminal is short-circuited, causing the dipoles to return to their original orientation, which generates a depolarization current flowing through the insulation [91]. PDC data collection, an offline technique, typically requires around 20,000 s, with 10,000 s allocated to each phase of polarization and depolarization [92]. Before performing PDC measurements on a transformer, it must first reach thermal equilibrium with its surroundings. If the transformer is online, the four terminals of the high- and low-voltage windings are connected for a sufficient duration to eliminate any residual charge. Afterward, two terminals from each winding are short-circuited to form the electrodes across which the DC excitation voltage is applied for the polarization current measurement [93]. Given the low magnitude of the currents, an electrometer is typically used to record the data [94]. The test setup for PDC and a typical PDC plot [39] are shown in Figure 9a and Figure 9b, respectively.

The mathematical formulation of the polarization and depolarization currents is presented in the following equations [52,84,91]:

(11) $$i_{p}(t)=C_{0}U_{0}\left[\frac{\sigma }{\epsilon_{0}}+f(t)\right]$$

Respectively, σ, $U_{0}$, $\epsilon_{0},\;C_{0}$, and f(t) represent the composite conductivity, applied DC voltage, vacuum permittivity, geometric capacitance of the oil–paper insulation, and dielectric response function of the oil–paper insulation. $C_{0}$ is defined as

(12) $$C_{0}=\frac{C_{m}}{\epsilon_{r}}$$

where $C_{m}$ denotes the capacitance between the transformer and the ground, and $\epsilon_{r}$ is the heterogeneous oil–paper insulation system effective relative permittivity. The depolarization current is given as

(13) $$i_{d}(t)=C_{0}U_{0}\left[f\left(t\right)-f(t+t_{c})\right]$$

If $t+t_{c}$ is sufficiently high, then $f(t+t_{c})≅0$. Therefore, from (10) and with the help of only the depolarization current, the dielectric response function can be computed as

(14) $$f\left(t\right)\approx \frac{-i_{d}(t)}{C_{0}U_{0}}$$

Additionally, during the polarization phase, both dipole relaxation and DC conduction currents are present, whereas in the depolarization phase, only the dipole relaxation current is observed. The average DC conductivity of the composite insulation can be determined by calculating the difference between the dipole relaxation currents (polarization and depolarization currents) of the composite insulation. This can be expressed by rewriting (8) and (10) as

(15) $$\sigma \approx \frac{\epsilon_{0}}{C_{0}U_{0}}\left(i_{p}\left(t\right)-i_{d}(t)\right)$$

**Figure 10:**
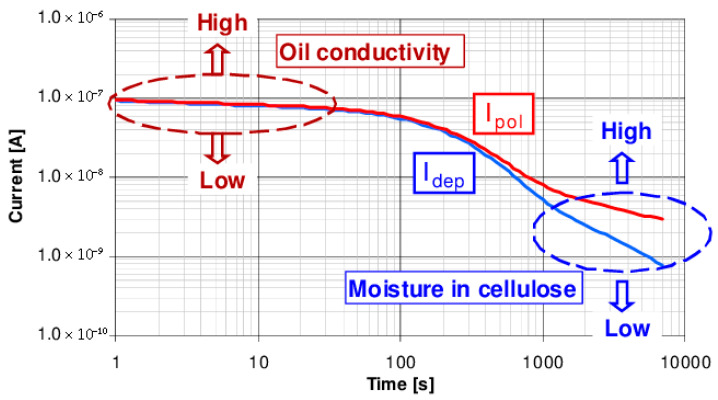
Diagnostics of Oil-Paper-Insulations Using Relaxation Currents [57].

The typical PDC curve, depicted in Figure 10 offers valuable insights into the conductivity, aging, and moisture content of insulation. In the time range t<100 s, the curve is predominantly influenced by conductivity, with the current increasing proportionally to its value. Beyond 100 s, the effects of oil properties, aging, and insulation geometry become more apparent, and variations in moisture content are noticeable after 1000s [95]. Although not a dedicated standard, the IEC/IEEE Guide for the Statistical Analysis of Electrical Insulation Breakdown Data [96] can be of use when it comes to PDC measurement and data analysis.

One of the primary advantages of the PDC diagnostic technique is that, from the PDC data, several other aging-sensitive condition monitoring parameters can be estimated or directly calculated. These parameters include tan (δ), %pm (paper moisture), dielectric adsorption ratio (DAR), and PI. Both tan (δ) and %pm can be estimated from the derived Debye model based on PDC data, as these parameters are related to the transfer function zeros, as follows:

(16) $$tan\delta =0.4423+0.0457\times \ln\left(\left|Z1\right|\right)$$

(17) $$\%pm=1.718+0.418\times \ln\left(\left|Z1\right|\right)$$

For DAR and PI, they can be derived from the IR at various time intervals, as discussed in Section 3.5, and obtained directly from the measured PDC data.

However, a significant concern with PDC measurement is its longer duration, which leads to extended downtime, posing a challenge for utilities since the technique is offline. Many researchers have attempted to address this issue. For example, in [88], the authors proposed the use of neural networks to forecast and predict the complete PDC curve from partial data recorded over shorter time spans, thus reducing measurement time. It was found that PDC data recorded for 600 s were sufficient to accurately forecast the full 10,000 s curve. The impact of temperature variations on the dielectric response of oil–paper insulation was comprehensively studied in [97]. Other areas of interest include modeling the dielectric response in PDC [98,99].

Further research into PDC could explore methods to compensate for temperature effects on the dielectric response function, as well as the impact of de-trapped charges (de-trapping current) during the PDC measurement phase. Many insulation-based models assume that the polarization phase is free of de-trapped charges; however, the authors in [92] argue that this assumption is unreasonable, supporting their findings with data from operational transformers and recommending further exploration in this area. As PDC is also affected by field noise, research into noise-resilient methodologies and advanced filtering techniques is a promising direction. Additionally, integrating AI techniques, such as artificial neural networks (ANNs), as demonstrated in [100], but applied to alternative datasets rather than PDC, along with advanced forecasting methods, could significantly reduce PDC measurement time. This advancement would provide substantial benefits to the scientific community.

#### 3.7.3. Frequency Domain Spectroscopy (FDS)

In frequency domain spectroscopy (FDS), also known as dielectric frequency domain response (FDR), a sinusoidal voltage is applied to a test specimen, and the resulting current through the test object is measured. FDR is used to evaluate the dielectric response of composite oil–paper insulation across frequencies ranging from 100 μHz to 1 kHz. The frequency range may vary based on the moisture content in the insulation. However, prominent resonance effects and impure dielectric responses limit the maximum frequency to 1 kHz [57,101]. The complex insulation system’s response is typically represented as a plot of the loss factor (tanδ) or capacitance versus frequency [102,103].

If the external voltage U(t) is applied to the test object, the current in time domain flowing through the test insulating object of geometrical capacitance $C_{0}$ is given as ([98])

(18) $$I\left(t\right)=C_{0}\left(\frac{\sigma_{dc}}{\varepsilon_{0}}+\varepsilon_{\infty }\delta \left(t\right)+f\left(t\right)\right)U(t)$$

Here, $C_{0}$ represents the geometrical capacitance of the test system. From (15), it is evident that the measured current is determined by the permittivity (ε), DC conductivity ($\sigma_{dc}$), and dielectric response function f(t) of the material under test.

The current in frequency domain passing through the dielectric material in response to a sinusoidal, time-varying electric field can be calculated by applying the Fourier transform to the given equation [98,101,104]:

(19) $$I\left(\omega \right)=j\omega C_{0}U_{0}\underset{\varepsilon^{\prime }(\omega )}{\underbrace{\left[\left(\varepsilon_{\infty }-\chi^{\prime }\left(\omega \right)\right)\right.}-}\underset{\varepsilon^{\prime \prime }(\omega )}{\underbrace{\left.\left(\frac{\sigma_{dc}}{\varepsilon_{0}\omega }+\chi^{\prime \prime }\left(\omega \right)\right)\right]}}$$

(20) $$I\left(\omega \right)=j\omega C_{0}U_{0}\left\{\varepsilon^{\prime }\left(\omega \right)-\varepsilon^{\prime \prime }(\omega )\right\}$$

where $C_{0},\;U_{0}$, $\sigma_{dc}$, $\varepsilon^{\prime }$, $\varepsilon^{\prime \prime }$, $\chi^{\prime }$, and $\chi^{\prime \prime }$ represent the geometric capacitance, DC conductivity, real permittivity, imaginary permittivity, real susceptibility, and imaginary susceptibility, respectively.

The real and imaginary components of the measured current correspond to the current in phase and out of phase with the applied voltage. The imaginary component represents energy losses, which comprise resistive losses from conduction current and dielectric losses from electric polarization. When the geometry of the test specimen is unknown, the dielectric response is conveniently expressed as the loss factor (tanδ) defined as the ratio of the imaginary permittivity ($\varepsilon^{\prime \prime }(\omega )$) to the real permittivity ($\varepsilon^{\prime }(\omega )$) as ([101]):

(21) $$tan\delta =\frac{\varepsilon^{\prime \prime }(\omega )}{\varepsilon^{\prime }(\omega )}$$

**Figure 11:**
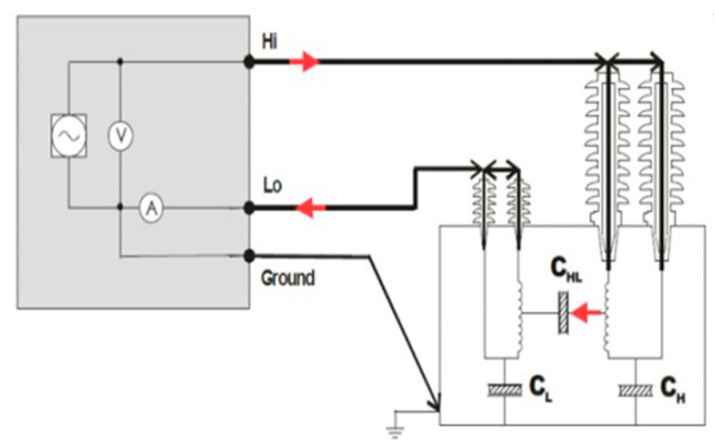
FDS measurement circuit [101].

For FDS measurements, the high- and low-voltage bushings of the power transformer are interconnected (see Figure 11). The FDS measurement is then conducted using a low voltage, typically 140 V RMS, to minimize voltage-dependent effects at low frequencies. However, higher voltages may be employed in environments with significant interference [101,105]. The primary focus is on measuring the main insulation between the high-voltage (HV) and low-voltage (LV) windings. Also, the insulation of the winding to ground, such as high voltage to ground ($C_{H}$) and low voltage to ground ($C_{L}$), are additional measurements typically performed [102]. The IEC 60076-18 [31] and IEEE 62-1995 [106] standards are guides for FDS measurement.

**Figure 12:**
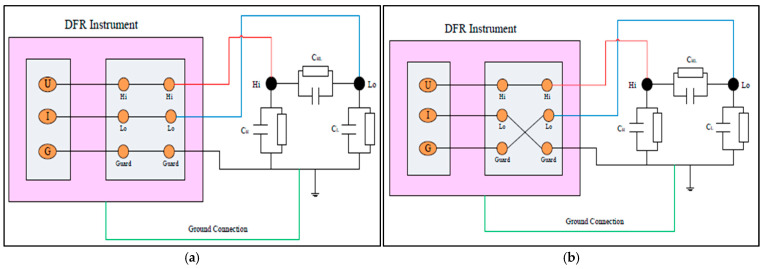
(**a**) GST mode for FDS; (**b**) UST mode for FDS [101].

Measurement Configurations:

The general concept of an FDS test can be performed in two modes:

1. Un-Grounded Specimen Test (UST) Mode: This mode assesses the condition of the insulation between the LV and HV windings by measuring the current flowing through the HV winding to the LV winding, thus completing the circuit path. In this configuration, only the current passing directly from HV to LV contributes to the results (see Figure 12b) [101,102].
2. Grounded Specimen Test (GST) Mode: This mode also evaluates the insulation condition between the LV and HV windings. The circuit current flows from the HV winding to the LV winding [101]. In this measurement, only the current flowing from HV to LV winding is considered (see Figure 12b) [102].

**Figure 13:**
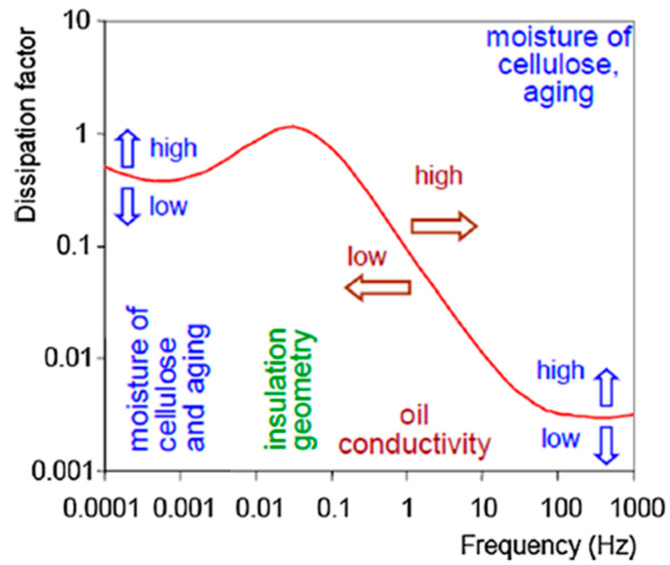
Frequency dielectric response of paper–oil insulation [23,57].

The FDS method effectively differentiates the effects of oil and paper insulation, as clearly illustrated in Figure 13. The typical S-shaped curve observed in FDS is primarily influenced by four factors: moisture, oil conductivity, insulation geometry, and temperature. As depicted in Figure 13, each of these factors influences the curve in distinct ways. For instance, an increase in oil conductivity shifts the curve towards higher frequencies, while insulation geometry contributes to the “hump” in the curve. Moisture content in cellulose and aging tend to shift the curve upwards towards higher dissipation factor values, while increasing or decreasing moisture shifts the curve downward [57,107].

The influence of temperature on FDS is particularly critical when it comes to ensuring the accuracy and reliability of the test results. Therefore, significant research is focused on developing temperature compensation methods or generating master curves at various temperatures to be used as reference points [105]. Exploring temperature compensation techniques could be a promising avenue for further research. Additionally, the complex nature of interpreting the FDS curve calls for advanced tools like AI. In [108], the authors employed a genetic algorithm-supported vector machine (GA-SVM) to diagnose moisture in transformer insulation using FDS. According to [109], FDS is one of the most complex and cost-intensive diagnostic methods for power transformers. Research into more straightforward and cost-effective setups, or the development of portable solutions for on-site testing, would enhance the accessibility and practicality of this method. Based on a review of the various dielectric response analysis methods, the authors compared three techniques in Table 4.

### 3.8. Breakdown Voltage Test

The breakdown voltage (BDV) test, also known as the AC electric strength test, is a critical method for assessing the dielectric strength of transformer oils. This test evaluates the oil’s ability to withstand electrical stress, which is vital for ensuring the reliability of transformer insulation [110]. BDV is highly sensitive to contaminants such as water and solid particles, both of which significantly reduce the oil’s insulating properties. For clean, new, and dry insulating oils, typical BDV values are above 70 kV for mineral oils and esters, and above 50 kV for silicone fluids. However, contamination can cause substantial reductions in these values, making BDV an essential diagnostic tool for monitoring the condition of transformer oils [111].

**Figure 14:**
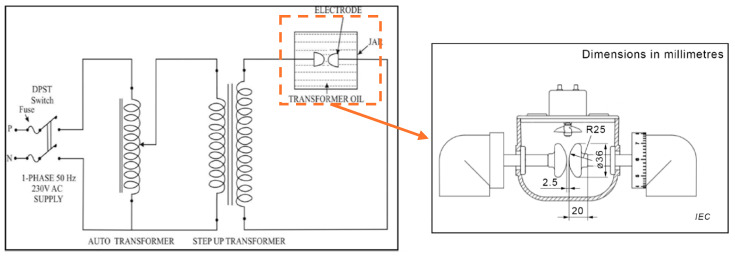
Breakdown voltage test circuit configuration [114].

The BDV test is usually conducted following standards such as IEC 60156 and ASTM D1816, typically at room temperature. The method involves applying a power-frequency voltage to the oil sample until a breakdown occurs between electrodes immersed in the oil. Standard test configurations use disc or spherical electrodes with gap distances ranging from 1 mm to 2.5 mm, depending on the applicable standard. Figure 14 illustrates a typical BDV test setup [112]. For example, the IEC 60156 standard specifies electrodes with diameters between 12.5 and 13 mm, while ASTM D877 mandates a minimum breakdown voltage of 30 kV for insulating oils used in transformers rated between 69 kV and 230 kV (refer to Table 5 [113]). In summary, the three standards relevant to BDV measurement are IEC 60156:2025 [114], ASTM D1816 [115], and ASTM D877 [116]. The main differences between these standards are the type and diameter of electrodes used. A higher BDV indicates superior insulating properties, but it is important to note that a high BDV does not necessarily imply the absence of contaminants [117].

Moisture and solid particles are the primary contaminants that reduce the BDV of transformer oils. Excess water significantly lowers the dielectric strength, potentially leading to static discharge, PD, and tracking. The presence of solid particles, particularly conductive ones like metals, carbon, and wet fibers, exacerbates the issue. These particles cause localized electrical stress, which can lead to the premature failure of transformer insulation. Therefore, identifying and counting particles is critical for a comprehensive condition assessment of the oil [118]. Additionally, when temperatures decrease, water may form emulsions, further lowering BDV, particularly in the presence of impurities [118,119].

To ensure accuracy, BDV tests are typically performed multiple times, with the average BDV value used to assess the oil’s condition. Advanced methodologies, such as cumulative Gaussian probabilities, are employed to better characterize BDV results. Based on the measured BDV, the oil is classified into three categories: “poor”, “fair”, “good”. For example, an average BDV of 55 kV indicates “good” oil condition, while lower values may suggest the need for further analysis and potential oil treatment. In-service transformers generally require a minimum BDV of 30 kV when measured with a 2.5 mm gap, although the exact criteria can vary depending on the transformer’s voltage rating [113,120].

In addition to laboratory tests, BDV measurements can be performed on-site in substations using portable equipment. This on-site capability facilitates the convenient and timely condition monitoring of transformer oils without the need to remove samples for laboratory testing. Portable BDV testing devices provide valuable data that can guide maintenance decisions, ensuring the continued safe operation of transformers [112,113].

While BDV testing can provide valuable insights into the condition of insulating oil, it is most effective when combined with other methods, such as dissolved gas analysis. Though BDV testing is quick for detecting contamination, it requires periodic testing and cannot identify specific faults [121]. A promising area for future research involves integrating BDV testing with other diagnostic techniques to enhance fault detection. The effects of particle type, size, and distribution on BDV are significant and warrant further study. Additionally, environmental factors like sampling conditions and temperature influence BDV results and quantifying these effects could improve diagnostic accuracy. The combined impact of moisture and particulate contamination on BDV is complex, and further research into their synergistic effects will provide better diagnostic tools.

### 3.9. Power Factor and Capacitance

**Figure 15:**
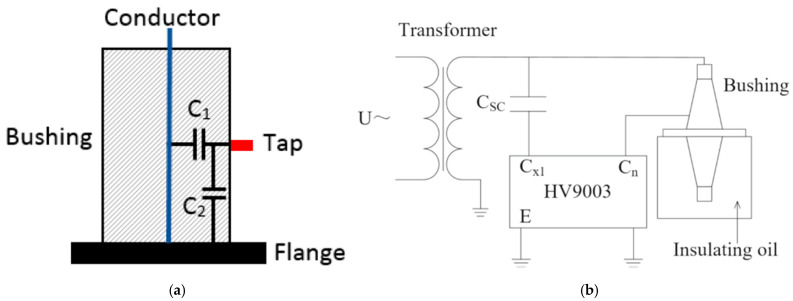
(**a**) Bushing equivalent capacitances [123]; (**b**) measurement test setup [122].

One of the methods used to diagnose the condition of high-voltage bushings is through the measurement of capacitance and power factor. These measurements are compared against benchmark data to assess the bushing’s condition. Transformer bushings can be modeled as a series of capacitors, with their electrical behavior characterized by two key capacitances (see Figure 15a): C1, the capacitance between the conductor and the dielectric dissipation factor (DDF) tap, and C2, the capacitance between the DDF tap and the ground. A test setup for high-voltage dielectric loss and capacitance measurement using an HV9003 high-voltage variable frequency anti-interference dielectric loss tester is shown in Figure 15b [122]. Bushings are vulnerable to issues such as oil leakage in oil-impregnated paper (OIP) bushings, moisture, and resin-bonded paper cracking. These abnormalities cause changes in the values of C1 and C2 throughout the bushing’s operational life. Consequently, capacitance measurement becomes a valuable diagnostic tool for assessing the condition of bushings [57,123].

This diagnostic approach involves monitoring the variation in PF and its dependence on capacitance values [124]. Many methods have been proposed for the real-time, online monitoring of bushings, facilitating continuous condition assessment. Research has shown that measuring PF and capacitance under operating voltage provides comprehensive insights into bushing performance. The PF reflects dielectric losses in the insulation, which are influenced by contamination and degradation of both solid and liquid insulating materials. Capacitance measurements, on the other hand, help detect physical defects, such as shorted capacitive layers or oil leaks, offering a complete view of the bushing’s integrity [125,126].

Diagnosis can be performed in three ways: by monitoring the trend of measured data over time, comparing the measurements to nameplate data or values taken during a healthy state (or from bushings of other phases), and using absolute limits set by standards. For instance, IEEE Std C57.19.01 TM-2017 [127] sets a PF limit of ≤0.5%. Some manufacturers also recommend that the PF, when corrected to 20 °C, should not exceed twice or thrice the benchmark value. Similarly, for capacitance, a deviation of 5–10% from the benchmark is commonly used as an action threshold, prompting further investigation or maintenance [125,126,128].

The trend in capacitance and power factor measurement is moving towards the online monitoring of these parameters. Several authors have presented methods to obtain capacitance (C) and dissipation factor (DF) values online. However, very few papers have been published on this method in the past decade, indicating a gap in recent research. Other diagnostic techniques, such as dissolved gas analysis [129,130], PD measurements [131,132], and FRA [130,133], can also be used to diagnose high-voltage bushings. These methods have gained popularity in recent years.

A potential improvement to capacitance and power factor diagnostics could involve the integration of AI models with online measurement systems. Another important research challenge is discriminating or eliminating the effects of stray capacitances on test results, as adjacent substation equipment can influence measurements. 

A summary of all diagnostic techniques, along with their associated research challenges, is presented in Table 6. For each method, the table provides a concise overview of the measurand, data analysis techniques, potential research directions, and relevant references.

## 4. Advances in AI/ML for Power Transformer Diagnosis

This section provides a brief discussion on the application of artificial intelligence (AI) and machine learning (ML) in power transformer diagnostics. For a more in-depth analysis, refer to [12,135,136]. Table 7 summarizes the use of AI across various diagnostic techniques. While multiple AI/ML algorithms have been applied in this field, Table 7 highlights that neural networks are the most widely used approach. Although AI/ML has been extensively applied to certain techniques, others have received little to no attention. Currently, there is no international standard guiding the application of AI in data analysis for power transformer diagnostics. Given the growing reliance on neural networks and other ML algorithms, establishing such a standard would be highly beneficial.

## 5. Instrumentation

The instrumentation required for performing the electrical diagnostic techniques discussed above varies significantly depending on the method employed. Even within the same diagnostic technique, variations may arise due to slight differences in methodologies formulated by different researchers. Additionally, various commercial products are available for the same diagnostic technique, leading to differences in experimental setups across literature. While the underlying theory of each technique remains consistent, several researchers have introduced innovative approaches that modify or complement the original theories to achieve optimal results and enhance other parameters.

Table 8 provides an overview of the fundamental instruments needed for each diagnostic technique presented and how they can be carried out, either online onsite or offline [149,150]. The cross (✗) and check (✔) symbols indicate non-applicability and applicability, respectively. It is important to note that additional instruments may be required in certain cases or to complete the experimental setup. The table also includes specific instruments used by various researchers. Furthermore, the specific international standards applicable to each diagnostic technique are summarized in the table to ensure completeness and provide a comprehensive reference.

Although different commercial instruments from various manufacturers may be employed, from a metrological perspective, each instrument must meet certain requirements to ensure reliable results and minimize measurement uncertainties. These requirements include high accuracy, stability, and a well-defined measurement range tailored to the specific diagnostic technique [151,152]. Calibration and traceability to international standards, such as those established by IEC or IEEE, are crucial for ensuring consistency across different studies. Other factors, such as signal-to-noise ratio, frequency response, and environmental robustness, must also be considered, particularly for field measurements where external conditions can impact readings [153].

When multiple instruments are used, and the acquired signal undergoes several stages of conversion, the combined uncertainty must be accounted for in the measurement process. The Guide to the Expression of Uncertainty in Measurement (GUM) is a valuable resource for this purpose [154]. Selecting the appropriate instrument involves not only choosing a recognized commercial product but also validating its performance against standard reference materials or established benchmarks to enhance the reliability and accuracy of the diagnostic process.

It is worth noting that offline techniques can either be performed onsite or offsite in the laboratory. For instance, offline electrical PD detection is performed offsite in the laboratory. Although online measurement is desirable by utilities, the criticalities of this approach limit its adoption in some cases. Online measurement techniques are usually influenced by uncontrolled inference such as stray electromagnetic fields, and environmental factors especially when the acquired signal is of low magnitude [155]. Additionally, some techniques require sending a signal (voltage, current, etc.) and when the equipment is in operation, this becomes a difficult task. In general, online methods often require sophisticated filtering and noise reduction techniques to extract meaningful data, adding complexity to the diagnostic process. Despite these challenges, online monitoring remains an attractive option due to its ability to provide real-time condition assessment without requiring equipment shutdown [156].

## 6. Discussion

The electrical diagnostic techniques discussed in Section 3 above do not represent a comprehensive list of all available methods for diagnosing the condition of power transformers. However, the methods covered in this review are among the most widely used. Several other techniques have been explored in the literature, including impulse voltage testing [157], induced voltage withstand test [158,159], excitation current test, transfer function measurement [160,161], transformer loading, OLTC dynamic resistance test [162,163,164], and leakage/short-circuit impedance test [50,165]. According to the advanced search algorithm applied for this review, which is limited to the last decade, these techniques have been studied to a lesser extent during this period. Despite their limitations, these methods remain valid for diagnosing power transformers. However, with the advent of emerging technologies and the evolution of traditional power systems, other methods, such as those presented in Section 3, have gained superiority in terms of performance, which is why current research is more focused on them.

**Figure 16:**
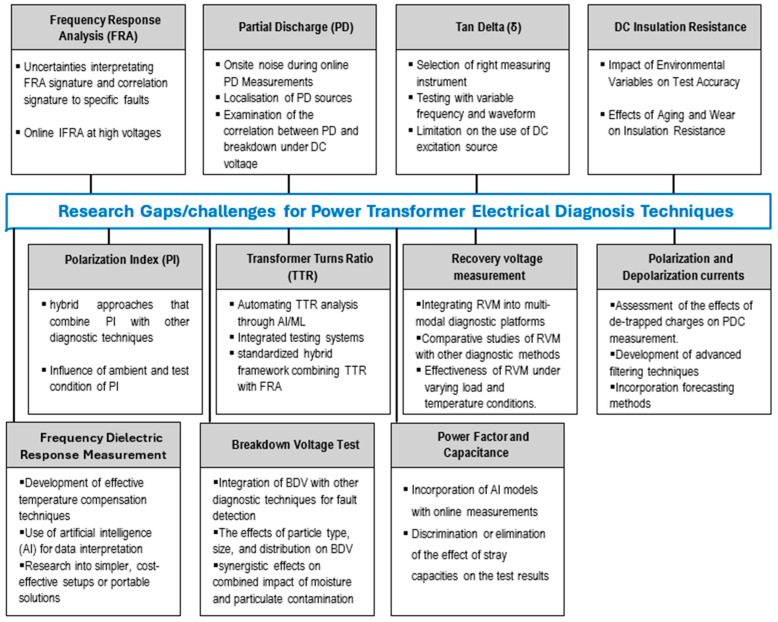
Research gaps/challenges for power transformer electrical diagnosis techniques.

Overall, electrical diagnostic methods are effective for monitoring the condition of power transformers and detecting faults. They can identify various electrical and mechanical faults, such as winding deformation, core movement, insulation failure, PD, loose core, moisture contamination, and bushing failure. In some cases, such as PD detection, electrical methods have proven to be highly sensitive, despite their limitations in other aspects. Figure 16 outlines the research challenges associated with each technique, highlighting gaps in literature that require further investigation to improve the effectiveness of these methods.

Over the last decade, there has been an increasing trend in the application of AI for diagnosing power transformers. In particular, several machine learning (ML) algorithms have been employed to analyze results obtained from various testing methods [50,135,136]. Driven by the need to reduce uncertainty and improve traditional methods that rely on expert judgment for analyzing trends and identifying abnormalities in recorded data, ML algorithms have become a prominent area of focus in literature. Some of the most widely used ML algorithms include artificial neural networks (ANNs), support vector machine (SVM), random forest (RF), k-nearest neighbors (KNN), decision tree (DT), deep learning (convolutional neural networks, long short-term memory (LSTM)), clustering algorithms (e.g., k-means, DBSCAN), and Bayesian networks. In some cases, ML algorithms complement human expertise rather than replace it, as it is believed that experts possess certain insights that AI cannot replicate.

Though not captured in this review, it is worth noting that machine learning has been extensively applied to the dissolved gas analysis (DGA) of power transformers. Based on the bibliography extracted using the advanced search algorithm presented in Section 2, over 50% of the papers focus on DGA, with many of these studies exploring the use of ML to analyze test data or compare the effectiveness of various ML algorithms [166,167]. However, there is also a growing use of ML in other electrical diagnostic methods, such as FRA, polarization depolarization current testing, and frequency domain spectroscopy.

Currently, there are no dedicated international standards specifically focused on the use of AI or machine learning (ML) for the diagnosis of power transformers. Many authors have raised concerns about the inconsistency in applying ML for transformer diagnosis, which often leads to challenges in replicating results. Given the widespread use of ML in diagnosing one of the most critical components of the power network, it is high time for the development of an international standard to guide the application of AI in this field. A new era demands the establishment of a new standard!

## 7. Conclusions

This review offers a comprehensive analysis of electrical diagnostic techniques for medium-voltage assets, focusing on various methods, instrumentation, and emerging research challenges. Condition monitoring and fault diagnosis are essential for ensuring the reliability and longevity of power equipment, with electrical methods being among the most precise and effective solutions for fault detection. The study highlights a range of diagnostic techniques, including frequency response analysis, partial discharge measurement, dielectric dissipation factor, transformer turn ratio, and dielectric response analysis. Each technique offers distinct advantages and limitations, underscoring the importance of selecting the appropriate diagnostic tools based on specific application requirements.

In addition to a detailed review of widely used electrical diagnostic methods, this paper consolidates the key instruments required to establish the test circuits for reference. In addition, the international standards relevant to each of the methods have been summarized in this review. The metrological requirements for these instruments, necessary for reliable performance, are also briefly discussed. The application of artificial intelligence (AI) and machine learning (ML) in power transformer diagnostics has been discussed, along with recommendations for establishing an international standard. While electrical methods for power transformer diagnosis are effective in condition monitoring and fault detection, they continue to face challenges. Several research gaps and future directions have been identified to enhance the methods discussed. Overall, this research provides a holistic overview of electrical diagnostic techniques, addressing various methods, instrumentation, and potential research directions for early-career researchers venturing into the electrical diagnosis of power transformers.

## Figures and Tables

**Table 1:** Voltage range for insulation resistance [56,68].

Winding Rated Voltage	Insulation Resistance Test Voltage $\left(V_{dc}\right)$
<1000	500
1000–2500	500–1000
2501–5000	1000–2500
5000–12,000	2500–5000
>12,000	5000–10,000

**Table 2:** Minimum pi values based on thermal class of insulator [56].

Thermal Class Rating	Minimum PI Value
Class 105 (A)	1.5
Class 130 (B) and above	2.0

**Table 3:** Different PI testing modes.

Test Mode	1 min	10 min	PI
Primary–GND	${IR}_{\left(p-g\right)1}$	${IR}_{\left(p-g\right)10}$	$\frac{{IR}_{\left(p-g\right)10}}{{IR}_{\left(p-g\right)1}}$
Secondary–GND	${IR}_{\left(s-g\right)1}$	${IR}_{\left(s-g\right)10}$	$\frac{{IR}_{\left(s-g\right)10}}{{IR}_{\left(s-g\right)1}}$
Tertiary–GND	${IR}_{\left(t-g\right)1}$	${IR}_{\left(t-g\right)10}$	$\frac{{IR}_{\left(t-g\right)10}}{{IR}_{\left(t-g\right)1}}$
Primary–Secondary	${IR}_{\left(p-s\right)1}$	${IR}_{\left(p-s\right)10}$	$\frac{{IR}_{\left(p-s\right)10}}{{IR}_{\left(p-s\right)1}}$
Primary–Tertial	${IR}_{\left(p-t\right)1}$	${IR}_{\left(p-t\right)10}$	$\frac{{IR}_{\left(p-t\right)10}}{{IR}_{\left(p-t\right)1}}$
Secondary–Tertiary	${IR}_{\left(s-t\right)1}$	${IR}_{\left(s-t\right)10}$	$\frac{{IR}_{\left(s-t\right)10}}{{IR}_{\left(s-t\right)1}}$

**Table 4:** Comparison between dielectric response analysis (DRA) techniques.

Technique	Method	Advantages	Disadvantages	Domain
Recovery voltage measurement	Application of DC voltage with increasing charging and discharging time while maintaining $t_{c}/t_{d}=2$.	Simple and non-destructive.	Time consuming. Affected by environmental conditions (e.g., temperature).	Time domain
Return voltage measurement	Application of single charging DC voltage in the kV range.	Highly resistant to interference from external electric fields in the surrounding environment. Fast diagnosis.	Experienced experts are required, as RVM spectra can be challenging to differentiate from the combined effects of oil and paper insulation.	Time domain
Polarization depolarization current (PDC)	Application of DC voltage and measurement of polarization and depolarization current using an electrometer over a period (typically 20,000 s).	Other diagnostic parameters such as polarization index (PI), dissipation factor (%tanδ), dielectric adsorption ratio (DAR), paper moisture (%pm), etc., can be determined by analyzing the PDC data.	PDC data affected by de-trapped current. PDC measurement is affected by low-frequency field noise and temperature variation. Time-consuming, and thus, temperature variation can affect measurement.	Time domain
Frequency domain spectroscopy (FDS)	A sinusoidal voltage with frequencies ranging from 100 μHz to 1 kHz is applied to a test specimen, and the resulting current through the test object is measured	The dielectric frequency response method enables the direct evaluation of heterogenous (paper–oil) insulation by distinguishing between the effects of oil and paper insulation.	Interpreting and comparing FDS output curves is a complex task that demands specialized expertise in the field.	Frequency domain

**Table 5:** Limit value of dielectric breakdown voltage according to IEC60156 standard [55].

Method/Standard	Limit Value for Equipment with Voltage in the Range: 69–230 kV
ASTM D877	≥30 kV
ASTM D1816 (0.04)	≥28 kV
ASTM D1816 (0.08)	≥47 kV

**Table 6:** Summary of electrical diagnosis techniques.

Data Acquisition		Data Analysis/ Diagnosis Methods	Research Gaps/Challenges	Refs.
Detection Methods/Tests	Measurand			
Frequency Response Analysis (FRA)	Impedance, admittance or transfer function vs. frequency	Comparison of measured FRA signature with its healthy state, sister transformer, or other phases FRA signature of the transformer, statistical methods, ML, AI	Uncertainties interpretating FRA signature and correlation signature to specific faults Online IFRA at high voltages	[11,24,28,29,35]
Partial Discharge	Apparent Charge (pC)	Expert analysis of PRPD and TRPD spectrums, advanced signal processing techniques, such as discrete wavelet transform (DWT), AI (neutral networks, decision trees, etc.)	Onsite noise during online PD Measurements Localization of PD sources Examination of the correlation between PD and breakdown under DC voltage	[16,42,45,48,50,134]
Tan Delta	Dissipation factor/currents capacitance	Analysis of dielectric responses curves (frequency vs. tanδ), comparison of measures values with standards	Selection of right measuring instrument Testing with variable frequency and waveform Limitation on the use of DC excitation source	[56,58,62,63]
DC Insulation Resistance	Resistance (ohm)	Guidelines from standards Statistical methods	Impact of Environmental Variables on Test Accuracy Effects of Aging and Wear on Insulation Resistance	[18,56,71,112]
Polarization Index	Current	Guidelines from standards Statistical methods	hybrid approaches that combine PI with other diagnostic techniques Influence of ambient and test conditions of PI	[56,66,73,74]
Transformer Turn Ratio (TTR)	Current	Guidelines from standards Comparison of results with nameplate data	Automating TTR analysis through AI/ML Integrated testing systems Standardized hybrid framework combining TTR with FRA	[18,34,57,75]
Recovery/Return Voltage Measurements (RVMs)	Voltage	Comparison of the return voltage curve with the curve from the maxwell model Using the time constants $(\tau_{1}$ and $\tau_{2})$ and the r-factor as diagnosis tools	Integrating RVM into multi-modal diagnostic platforms Comparative studies of RVM with other diagnostic methods Effectiveness of RVM under varying load and temperature conditions Quantification of the effects of aging and moisture of RVM	[57,84,86]
Polarization Depolarization Current (PDC)	Current	Modeling the monotonically decreasing dielectric response using various insulation models (e.g., Debye model)	Methods for compensating for temperature effects on the dielectric response function Assessment of the effects of de-trapped charges (de-trapping current) on PDC measurement Development of advanced filtering techniques to mitigate the impact of field noise on PDC measurements Incorporation of advanced forecasting methods to reduce PDC measurement time	[88,94,97,98]
Frequency Domain Spectroscopy (FDS)	Current/tanδ	Time and frequency domain analysis; fitting models	Development of effective temperature compensation techniques Use of AI for data interpretation Research into simpler, cost-effective setups or portable solutions	[98,101,102,104,107]
Breakdown Voltage Tests	Voltage (kV)	The use of cumulative Gaussian probabilities Guidelines from Standards	Integration of BDV with other diagnostic techniques for fault detection The effects of particle type, size, and distribution on BDV Synergistic effects on combined impact of moisture and particulate contamination on BDV	[112,113,118,119,120]
Power Factor and Capacitance	Capacitance PF	Monitoring the trend of measures Comparison of measurement results with name plate data healthy state measurement The use of absolute limits imposed by standards	Incorporation of AI models with online measurements Discrimination or elimination of the effect of stray capacities on the test results	[125,126,128]

**Table 7:** Application of AI/ML for power transformer diagnosis.

Diagnosis Technique	Implementation of AI/ML	Results	Refs
Frequency response analysis (FRA)	This paper applied clustering analysis and cross-correlation methods to interpret frequency response analysis (FRA) data for diagnosing power transformer winding faults. It uses statistical clustering techniques to group different types of faults (short circuits, axial displacement, and radial deformation) and cross-correlation to measure the similarity between healthy and faulty transformer states.	The proposed approach successfully classified and diagnosed various winding faults with high accuracy. The clustering method provided a systematic way to distinguish different fault types, reducing reliance on expert judgment.	[137]
	This study combines logistic regression, discrete wavelet transform (DWT), and artificial neural networks (ANNs) to enhance fault detection in transformer windings. DWT is used for feature extraction, logistic regression selects the most effective wavelet bases, and ANN classifies different fault types.	The proposed model achieved a 97% accuracy rate in detecting transformer faults and reduced misclassification to 2.9%.	[138]
	The paper implements unsupervised machine learning using the k-means clustering method to classify power transformer states into groups based on failure probability. Supervised machine learning techniques, including artificial neural networks (ANNs) and adaptive neuro-fuzzy inference systems (ANFISs), are used to detect fault severity in power transformers of different lifetimes.	The k-means clustering method effectively groups transformers based on their health state, while ANN and ANFIS provide accurate fault severity detection.	[139]
	The paper proposes a data augmentation technique using a Conditional Wasserstein Generative Adversarial Network with Gradient Penalty (Conditional-WGAN-GP) to generate synthetic frequency response analysis (FRA) data. This augmented dataset is used to train fault diagnosis models, including support vector machines (SVMs), to detect winding deformation faults in transformers.	The proposed data augmentation technique significantly improves the accuracy of fault diagnosis models, with an average improvement of 5% compared to baseline models.	[140]
Partial discharge (PD)	The study proposes using an artificial neural network (ANN) combined with Cepstrum analysis to classify multiple partial discharge (PD) sources under noisy conditions. The ANN is trained on features extracted from PD signals using various methods, including discrete wavelet transform (DWT) and discrete Fourier transform (DFT), and then tested with noisy signals.	The Cepstrum-ANN method demonstrated the highest classification accuracy compared to other feature extraction techniques when classifying PDs under noisy conditions.	[141]
	The authors applied multiple machine learning techniques including support vector machine (SVM), k-nearest neighbors (kNN), naïve Bayes, random forest, and probabilistic neural network (PNN) to classify different types of PDs in paper–oil insulation. Feature extraction is performed using the power spectral density (PSD) of acoustic emission (AE) signals.	The SVM classifier with a polynomial kernel achieved 100% accuracy, while kNN also performed well. Random forest and naïve Bayes classifiers achieved over 97% accuracy, demonstrating the effectiveness of ML for PD identification.	[142]
	This study combines k-nearest neighbors (kNN) for imputing missing data and support vector machine (SVM) for classifying and localizing PD sources in power transformers. The dataset used is based on dissolved gas analysis (DGA), where kNN helps handle missing values before SVM processes the classification.	The combination of kNN and SVM significantly improved accuracy in PD classification and localization compared to traditional approaches. The study showed that handling missing values properly led to higher precision in PD diagnosis.	[143]
	The paper compares several machine learning and deep learning models, including support vector regression (SVR), back-propagation neural networks (BPNNs), convolutional neural networks (CNNs), and extreme gradient boosting (XGBoost), for the three-dimensional localization of PDs inside power transformer tanks. The models use electric field sensor data to predict PD locations.	CNN outperformed other methods in terms of localization accuracy, followed by XGBoost and SVR. The study found that using a single electric field sensor with ML techniques can provide high-precision PD localization, reducing the need for multiple sensors.	[41]
Tan delta	The authors proposed ANN model trained with a hybrid meta-heuristic algorithm called the particle swarm-based crow search algorithm (PS-CSA) to predict the aging of transformer insulation oil. The model uses input parameters including tan delta. The PS-CSA algorithm optimizes the weights of the ANN to minimize the error between predicted and actual aging outcomes.	The PS-CSA-ANN model outperforms LM-ANN, PSO-ANN, and CSA-ANN, showing significant improvements in error metrics like RMSE, MAE, and SMAPE. It achieves 49.6% lower RMSE than LM-ANN, 15.3% lower than FF-ANN, and 26.9% lower than CSA-ANN at a 25% learning rate.	[144]
IR and PI	The paper employs computational optimization techniques, specifically the hill-climbing algorithm combined with the 1/5th success rule, to refine the analysis and classification of power transformer insulation resistance.	The optimized method achieved an 88.9% accuracy rate in classifying insulation resistance conditions of power transformers.	[73]
TTR	No applicable papers (to the author’s knowledge).	Not applicable.	---
RVM	No applicable papers (to the author’s knowledge).	Not applicable.	---
PDC	The paper uses a neural network (NN) model to forecast polarization current (PDC) and reduce measurement time. The model predicts long-duration PDC data using only a short-duration sample, minimizing the need for extended testing while maintaining accuracy.	The NN model effectively forecasts polarization current, reducing measurement time from several hours to just 10 min while maintaining diagnostic accuracy.	[88]
	A residual LSTM model is used to forecast polarization current, reducing the need for lengthy PDC measurements. The model incorporates spatial shortcut connections to improve learning efficiency and is compared against LSTM, Attention-LSTM, GRU, and CNN. Monte Carlo dropout is used for uncertainty estimation.	The residual LSTM outperforms other models, achieving the lowest error metrics and the least uncertainty in forecasts.	[145]
FDS	The paper proposes a GA-SVM model for diagnosing moisture in transformer insulation using frequency domain spectroscopy (FDS). It introduces a novel method to predict FDS curves with limited data, improving classification accuracy.	The GA-SVM model achieves 99.15% classification accuracy, outperforming standard SVM and particle swarm optimization SVM (PSO-SVM).	[108]
	The paper uses machine learning (ML) models to classify moisture levels in transformer paper insulation based on frequency domain spectroscopy (FDS) data. It evaluates the support vector machine (SVM), artificial neural network (ANN), and k-nearest neighbors (KNN) algorithms for their ability to classify insulation moisture levels.	SVM outperformed ANN and KNN, achieving the highest classification accuracy and fastest training speed.	[146]
BDV	The paper develops an artificial neural network (ANN) model to predict the breakdown voltage (BDV) of transformer oil, considering the effects of barriers and contamination. The ANN model is trained on 784 experimental samples with various parameters such as electrode configurations, barrier properties, and temperature.	The ANN model achieves a prediction accuracy of 98.4% for training and 97.34% for testing, demonstrating its high reliability in estimating BDV.	[147]
	The study integrates genetic algorithm-optimized artificial neural networks (GA-BP-ANNs) and partial least squares regression (PLS-R) to predict the breakdown voltage of transformer oils using ATR-FTIR spectroscopy data. The GA optimizes feature selection, improving the ANN’s predictive performance.	The GA-BP-ANN model outperforms PLS-R, achieving a higher correlation coefficient (R^2^ = 0.9891) and lower RMSEP (0.2874), making it a highly accurate tool for oil condition monitoring.	[148]
PF and capacitance	No applicable papers (to the author’s knowledge).	Not applicable.	---

**Table 8:** Instrumentation for various diagnostic methods.

Diagnosis Method	Instruments Required	Online	Offline	Standard(s)
Sweep Frequency Response Analysis (SFRA)	ISA SFRA 5000 test instrument wide bandwidth current and voltage sensors	X	✓	IEC 60076-18 IEEE C57.149-2012
Impulse Frequency Response Analysis (IFRA)		✓	✓	IEC 60076-18 (not dedicated)
Partial Discharge	MPD600 system High-Frequency Current Transformers (HFCT)	✓	✓	IEC 60270
Tan Delta	Doble M4100 Omicron’s DIRANA Current and voltage instrument transformers	✓	✓	IEC 60851-5:2008
DC Insulation Resistance Polarization Index	Megger MIT 1525 DC voltage generator Current and voltage ITs	X	✓	IEC 60076-3:2013
Transformer Turn Ratio (TTR)	AC voltage generator Voltage instrument transformer	X	✓	IEEE C57.12.90-2021 IEC 60076-1:2013
Recovery/Return Voltage Measurements (RVMs)	RVM 5462b Recovery Voltage Meter Electrometer	X	✓	Not applicable
Polarization Depolarization Current (PDC)	Electrometer (e.g., Keithley 6517B Electrometer)	X	✓	Statistical Analysis) (not dedicated)
Frequency Domain Spectroscopy (FDS)	IDAX-300 insulation diagnostic analyzer	X	✓	IEC 60076-18 IEEE 62-1995
Breakdown Voltage Tests	Standard test electrodes made of disc or spherical electrodes	✓	✓	IEC 60156:2025 ASTM D1816 ASTM D877
Power Factor and Capacitance	M4000 insulation analyzer	✓	✓	IEC 60076-21-2011

## Abbreviations

The following abbreviations are used in this manuscript:

FRA	Frequency Response Analysis
SFRA	Sweep Frequency Response Analysis
IFRA	Impulse Frequency Response Analysis
AI	Artificial Intelligence
PD	Partial Discharge
UHF	Ultra-High Frequency
IR	Insulation Resistance
DAR	Dielectric Absorption Ratio
PI	Polarization Index
TTR	Transformer Turn Ratio
DRA	Dielectric Response Analysis
RVM	Recovery Voltage Measurement
PDC	Polarization Depolarization Currents
FDS	Frequency Domain Spectroscopy
BDV	Breakdown Voltage
PF	Power Factor
DDF	Dielectric Dissipation Factor
IEC	International Electrotechnical Commission
IEEE	Institute of Electrical and Electronics Engineers
